# Distinguishing neuroendocrine tumor grade 3 from neuroendocrine carcinoma in gastroenteropancreatic high-grade neoplasms

**DOI:** 10.1530/EO-25-0065

**Published:** 2025-12-19

**Authors:** Zandra Ankers, L Samuel Hellgren, Adam Stenman, Jan Zedenius, C Christofer Juhlin

**Affiliations:** ^1^ Department of Pathology and Cancer Diagnostics, Karolinska University Hospital, Stockholm, Sweden; ^2^ Department of Oncology-Pathology, Karolinska Institutet, Stockholm, Sweden; ^3^ Department of Breast, Endocrine Tumors, and Sarcoma, Karolinska University Hospital, Stockholm, Sweden; ^4^ Department of Molecular Medicine and Surgery, Karolinska Institutet, Stockholm, Sweden

**Keywords:** neuroendocrine tumor, neuroendocrine carcinoma, histology, immunohistochemistry, outcome

## Abstract

**Objective:**

High-grade neuroendocrine neoplasms (NENs) with a Ki-67 index >20% are currently classified as either grade 3 neuroendocrine tumors (NET G3) or neuroendocrine carcinomas (NEC). This study evaluates whether this subclassification is of diagnostic and prognostic significance.

**Design:**

We analyzed 98 high-grade NENs from 87 patients, categorized into NET G3 (*n* = 19), NEC (*n* = 76), and equivocal cases (*n* = 3).

**Methods:**

Histopathologic features, Ki-67 index, p53 and RB1 immunohistochemistry, growth patterns, necrosis, and somatostatin receptor type 2 (SSTR2) expression were assessed. Survival analyses were performed using Kaplan–Meier methods.

**Results:**

NEC patients were older, had higher Ki-67 indices, and more often displayed solid growth, while NET G3 showed organoid architecture. Aberrant p53 and RB1 expression were significantly more common in NECs (*P* = 0.001 and *P* < 0.001). NET G3 patients had significantly longer overall survival than NEC patients (*P* = 0.03). Within NECs, tumor origin influenced prognosis: colorectal NECs had better outcomes (HR = 0.494, *P* = 0.027), while cancer of unknown primary (CUP) was associated with worse survival (HR = 2.033, *P* = 0.022). Notably, 81% of NECs expressed SSTR2, suggesting potential diagnostic and therapeutic relevance.

**Conclusions:**

NET G3 and NEC show distinct clinicopathologic and prognostic profiles. Tumor origin offers additional stratification within NECs, while SSTR2 expression may inform targeted approaches. These findings underscore the need for nuanced classification and personalized management of high-grade NENs.

## Introduction

Gastroenteropancreatic neuroendocrine neoplasms (GEP-NENs) represent a diverse group of tumors originating from neuroendocrine cells within the gastrointestinal tract and pancreas (1, 2). These neoplasms are broadly classified into well-differentiated neuroendocrine tumors (NETs) and poorly differentiated neuroendocrine carcinomas (NECs), with significant differences in clinical behavior, prognosis, and therapeutic response (2, 3, 4, 5, 6, 7, 8, 9). In the 2017 WHO classification of pancreatic endocrine tumors, NET G3 was recognized as a distinct entity within high-grade neuroendocrine neoplasms. These tumors are well differentiated but exhibit a high proliferation rate, indicated by a Ki-67 labeling index greater than 20%. This distinction holds clinical significance, as NET G3 lesions generally have a better prognosis than NECs and may respond to chemotherapy regimens that are often ineffective in NEC patients (3, 10). Accurate differentiation between NET G3 and NEC in the GEP region is therefore suggested to hold clinical importance but remains challenging due to overlapping histological and immunohistochemical features (11, 12, 13, 14, 15). Diagnosis typically relies on morphological assessment, including growth pattern, cellular differentiation, and presence or absence of necrosis, combined with immunohistochemical markers such as the Ki-67 labeling index, p53, and RB1 (2). However, in routine clinical practice, the reproducibility and reliability of these parameters for distinguishing NET G3 from NEC remain uncertain.

Moreover, it is worth noting that a previous study has shown that 35% of pancreatic NET (PanNET) G3 cases may also have alterations in *TP53*, which further aggravates the role of the pathologist in distinguishing high-grade NENs into NET G3 and NEC (16). In accordance with this, two recently published studies have identified NETs with high-grade transformation exhibiting NEC-like co-alterations of TP53 and RB1 (17, 18). Thus, there may be an overlap between these entities, which may complicate the diagnostic process (19, 20).

In this study, we analyzed a cohort of NET G3 and NEC cases diagnosed at Karolinska University Hospital, a Swedish tertiary referral center with specialized endocrine pathology expertise. By limiting the study to cases evaluated at a single institution, we eliminated the interlaboratory variability commonly encountered in multicenter studies, where diagnostic consistency can be affected by differences in methodology and interpretation. Our study aimed to evaluate the histological and immunohistochemical features of NET G3 and NEC, focusing on their diagnostic relevance in routine practice. By correlating these parameters with patient outcome, we sought to identify key markers that improve the distinction between NET G3 and NEC.

## Materials and methods

### Tumor cohort, inclusion and exclusion criteria

All primary neuroendocrine neoplasms (NENs) and/or metastases of NENs diagnosed at Karolinska University Hospital, Stockholm, Sweden, between January 1, 2021, and July 21, 2024, were identified using electronic search functions with Systematized Nomenclature of Medicine (SNOMED) codes in our pathology database (M82463/6 for malignant neuroendocrine tumor/metastasis of malignant neuroendocrine tumor and M82493 for neuroendocrine carcinoma). The study period was limited to 2021–2024 to ensure the inclusion of cases diagnosed using updated diagnostic algorithms that incorporate the relatively new entity NET G3. This timeframe also guaranteed a standardized immunohistochemical work-up, which was not consistently implemented before 2021. Inclusion criteria encompassed cases of NET G3 and NEC originating from the gastroenteropancreatic (GEP) region or cases with an unknown primary tumor (NEN of unknown primary, NEN-UP). The histological work-up is based on morphological assessment as well as immunohistochemical verification of a neuroendocrine origin using antibodies targeting neuroendocrine markers of the first generation (chromogranin A and synaptophysin) as well as the second generation (ISL1 and/or INSM1). Exclusion criteria included neuroendocrine tumors grade 1 or 2, regardless of primary site, as well as neuroendocrine cancers originating outside the GEP region, such as small cell carcinoma of the lung, large cell neuroendocrine carcinoma of the lung, and rare cases of thymic, rhino-sinusoidal, and laryngeal NECs. Since the WHO classification was updated in 2022 regarding extrapancreatic NENs (2), we re-evaluated all extrapancreatic and CUP cases diagnosed in 2021–2022 to ensure a uniform diagnostic approach.

The study was approved by the Swedish Ethical Review Authority (Dnr 2012/305-31/1 and Dnr 2025-02219-02) and includes clinical and histopathological data from patients, the majority of whom are deceased. Although formal consent was not obtained from all individuals, all patients had been informed that their biopsy material could be stored in a biobank and used for research, with the option to opt out. Patients who actively declined biobank storage were excluded from the study. Accordingly, the requirement for informed consent was waived by the Swedish Ethical Review Authority.

### Histopathological variables

Data were collected from pathology reports in our reporting system. Information was gathered on sex, age at diagnosis, pathological diagnosis, primary tumor origin, whether the specimen was from a primary tumor or metastasis, type of specimen (biopsy or surgical), and metastatic site (either explicitly stated in the pathological referral or inferred from the specimen origin).

Histopathological data included the pattern of growth, categorized as solid/poorly differentiated, organoid/well differentiated, or a combination of both. Additional data included the presence or absence of tumor necrosis (defined as focal, comedo-like, or confluent ‘geographical’ tumor necrosis; coagulative-type necrosis was disregarded), the number of mitoses per 10 high-power fields (HPF) or per 2 mm^2^, the Ki-67 labeling index, and the number of PHH3-positive nuclei per 10 HPF or 2 mm^2^.

Immunohistochemical (IHC) data were also collected for somatostatin receptor type 2 (SSTR2), p53, RB1, ATRX, DAXX, gastrin, pancreatic islet hormones (insulin, glucagon, somatostatin, and pancreatic polypeptide), and serotonin. SSTR2 immunoreactivity was arbitrarily categorized as negative if less than 5% of tumor cells showed membranous staining; partially positive if 5–74% of tumor cells showed membranous staining; and positive if 75% or more of the tumor cells showed a membranous staining pattern.

### Clinical follow-up

A detailed clinical follow-up was conducted by reviewing patient files to collect relevant clinical data. All clinical data were systematically recorded and analyzed to assess patient outcomes. Variables included sex, age at diagnosis, primary tumor site (colorectum, pancreas, cancer of unknown primary (CUP), other), and metastatic site (liver, lung, lymph node, skeleton, or multiple sites). The type of specimen (biopsy, surgical resection) and specimen characteristics (primary tumor or metastasis) were also documented. Data regarding neoadjuvant or adjuvant chemotherapy, external radiation therapy, and radionuclide therapy were also recorded. Patient records were also examined to determine overall survival. Follow-up data were retrieved from clinical notes, radiology reports, and pathology records to assess survival status, recurrence, and disease progression. Overall survival was defined as the time from diagnosis to death or last follow-up, while disease-free survival was calculated from treatment to recurrence or last follow-up without recurrence.

### Statistical analyses

Statistical analyses were performed using IBM SPSS Statistics (version 29). Fisher’s exact test was applied to assess differences between groups for categorical variables, while the Mann–Whitney U test was used for continuous variables. Univariate and multivariate Cox regression analyses were performed to identify predictors of disease-specific mortality. Survival analysis was conducted using Kaplan–Meier curves, with log-rank tests to compare survival distributions between groups. Disease-specific mortality was used as an endpoint in these analyses. A *P*-value <0.05 was considered statistically significant.

## Results

### Tumor cohort

**Figure 1:**
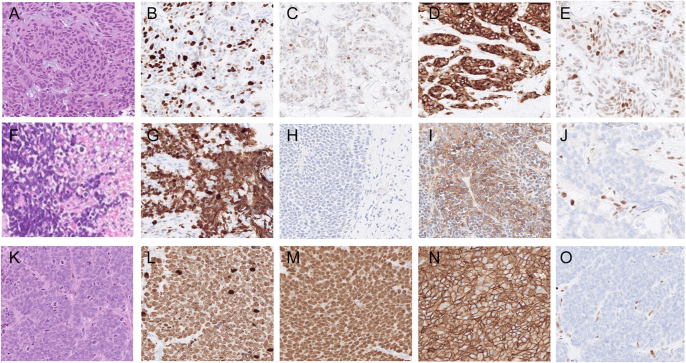
Histomorphological features and immunohistochemical findings in high-grade NENs. (A, B, C, D, E) Pancreatic NET (PanNET) G3. (A) Hematoxylin and eosin (H&E) staining displaying a nested growth pattern, indicating a well-differentiated tumor. (B) Ki-67 immunohistochemistry (IHC) with an index of 44%. (C) P53 IHC displaying a wild-type pattern, with partial nuclear expression of varying intensity. (D) IHC for SSTR2 with a diffuse expression. (E) RB1 IHC depicting variable immunoreactivity, consistent with a wild-type pattern. (F, G, H, I, J) Small cell neuroendocrine carcinoma (SC-NEC). (F) H&E staining depicting small cell features showing nuclear molding and heavily condensed chromatin; also note the tumor-specific necrosis. (G) Ki-67 IHC with a labeling index >90%. (H) The p53 IHC displaying a so-called ‘null-phenotype’, strongly indicating an underlying *TP53* mutation. (I) Focal membranous SSTR2 expression was evident. (J) Loss of RB1 IHC with retained stromal control, indicative of *RB1* gene mutation or deletion. (K, L, M, N, O) Large cell neuroendocrine carcinoma (LC-NEC). (K) H&E staining illustrating the high-grade morphology and solid growth. (L) Ki-67 IHC with a labeling index >90%. (M) The p53 IHC staining was diffusely positive, indicating an underlying *TP53* gene mutation. (N) The SSTR2 expression was strong and diffuse in this case. (O) Negative RB1 staining, suggestive of *RB1* gene inactivation. All images are magnified ×400.

A total of 319 cases were retrieved in our databases within the time period January 2021–July 2024 using the SNOMED search function. Of these 319 cases, 221 were excluded. Among these, 27 cases were classified as non-GEP NENs, 193 were diagnosed as NENs grade 1 or 2, and one case involved a patient assessed via second opinion who was already included in the cohort. Ultimately, 98 cases from 87 patients met the inclusion criteria, with 10 patients contributing more than one sample during the study period. Of these 98 cases, 95 were sub-classified as either NET G3 (*n* = 19) or NEC (*n* = 76), while 3 cases remained indeterminate (‘equivocal’). For the statistical analyses and reporting of the results, we only included each patient’s first sample containing a high-grade NEN, and therefore the final cohort includes 87 cases that were sub-classified into NET G3 (*n* = 16), and NEC (*n* = 68), and three equivocal cases. Examples of NET G3 and NEC lesions are provided in Fig. 1.

The majority of cases, 84 out of 87 (96.6%), were either primarily diagnosed or reviewed by one or several subspecialized endocrine pathologists in our department.

### Basic clinical and histopathological parameters

The main clinical parameters of the NET G3 and NEC groups are summarized in Table 1. Histological differentiation and predominant growth patterns were recorded for 67 out of 87 (77%) cases. Of the remaining 20 cases in which the growth pattern or differentiation was not explicitly described in the pathology report, 13 underwent morphological re-evaluation through consensus among three of the authors (CCJ, LSH, and ZA). Of these 13 cases, 11 were successfully classified based on growth pattern and/or degree of differentiation, while two remained inconclusive due to insufficient tumor material or biopsy artifacts.

**Table 1:** Summarized clinical data.

Parameter	NET G3 (*n* = 16)	NEC (*n* = 68)
Sex		
Female, *n* (%)	4 (25)	31 (45.6)
Male, *n* (%)	12 (75)	37 (54.4)
Age, median (range)	57 (36–84)	69 (35–90)
Primary		
Colorectum, *n* (%)	2 (12.5)	27 (39.7)
Pancreas, *n* (%)	5 (31.5)	14 (20.6)
CUP, *n* (%)	6 (37.5)	21 (30.9)
Other, *n* (%)	3 (18.75)	6 (8.8)
Metastatic site		
Liver, *n* (%)	13 (81.25)	26 (38.2)
Lung, *n* (%)	0	0
Lymph node, *n* (%)	2 (12.5)	11 (16.2)
Skeleton, *n* (%)	0	2 (2.9)
Other, *n* (%)	1 (6.25)	6 (8.8)
Multiple sites, *n* (%)	0	2 (2.9)
Not specified, *n* (%)	0	21 (31)
Type of specimen		
Biopsy, *n* (%)	14 (87.5)	53 (78)
Surgical resection, *n* (%)	2 (12.5)	15 (22)
Specimen characteristic		
Primary, *n* (%)	0	27 (39.7)
Metastasis, *n* (%)	16 (100)	40 (58.8)
Not specified, *n* (%)	0	1 (1.5)
Chemotherapy		
Before sampling, *n* (%)	2 (12.5)	12 (17.6)
After sampling, *n* (%)	15 (94)	48 (71)
External radiotherapy		
Before sampling, *n* (%)	0	2 (3)
After sampling, *n* (%)	1 (6.25)	13 (19)
Radionuclide therapy		
Before sampling, *n* (%)	2 (12.5)	3 (4.4)
After sampling, *n* (%)	4 (25)	4 (6)

NET G3, neuroendocrine tumor grade 3; NEC, neuroendocrine carcinoma; CUP, cancer of unknown primary.

The majority of samples, 70 out of 87 (80.5%), were biopsies. Among NET G3 and equivocal cases, all samples were taken from metastatic sites. In the NEC group, the majority of samples were also biopsies (*n* = 53), although a substantial proportion of the samples were acquired from the primary site (*n* = 27).

The NEC patients were older than NET G3 patients, exhibited a higher Ki-67 labeling index, and more frequently showed a solid growth pattern, whereas NET G3 patients more commonly displayed an organoid growth pattern (Tables 1 and 2). Furthermore, NEC tumors more often demonstrated aberrant immunohistochemical staining patterns for p53 and RB1 compared with NET G3 lesions. In our cohort, 25/38 (66%) of informative NECs displayed aberrant p53 immunostaining, while the number for NET G3 patients was 1/11 (9%). For RB1, the numbers with aberrant staining were 19/30 (63%) of informative NECs and 0/11 (0%) of NET G3 lesions. There was no statistically significant difference between groups with regard to patient sex or follow-up time.

**Table 2:** Summarized pathological data.

Parameter	NET G3 (*n* = 16)	NEC (*n* = 68)	*P*-value
Morphology/growth pattern			
WD/organoid, only, *n* (%)	9 (56)	9 (13.2)	<0.001*
PD/solid, only, *n* (%)	0	34 (50)	0.001*
Mixed, *n* (%)	7 (44)	18 (26.5)	
Unspecified, *n* (%)	0	7 (10.3)	
Ki-67 index, median (range)	29 (21–70)	84.5 (25–95.4)	<0.001*
Ki-67 index >55%, *n* (%)	1 (6)	64 (94)	<0.001*
Necrosis			<0.001*
Present, *n* (%)	0	35 (51.5)	
Absent, *n* (%)	16 (100)	19 (27.9)	
Not specified, *n* (%)	0	14 (20.6)	
p53			0.001*
Wild-type, *n* (%)	10 (62.5)	13 (19.1)	
Aberrant/mutated, *n* (%)	1 (6.25)	25 (36.8)	
Not specified, *n* (%)	5 (31.25)	30 (44.1)	
RB1			<0.001*
Retained, *n* (%)	11 (68.75)	11 (16.2)	
Lost/mutated, *n* (%)	0	19 (27.9)	
Not specified, *n* (%)	5 (31.25)	38 (55.9)	
ATRX			1.000
Retained, *n* (%)	4 (25)	10 (14.7)	
Lost/mutated, *n* (%)	0	2 (2.9)	
Not specified, *n* (%)	12 (75)	56 (82.4)	
DAXX			1.000
Retained, *n* (%)	5 (31.25)	12 (17.6)	
Lost/mutated, *n* (%)	0	1 (1.5)	
Not specified, *n* (%)	11 (68.75)	55 (80.9)	

NET G3, neuroendocrine tumor grade 3; NEC, neuroendocrine carcinoma; WD, well differentiated; PD, poorly differentiated; RB1, retinoblastoma; ATRX, alpha-thalassemia/mental retardation syndrome X-linked; DAXX, death domain-associated protein.

* Significant *P*-value.

NET G3 lesions were most frequently found as tumors of unknown origin (*n* = 6) or in the pancreas (*n* = 5), while NECs most commonly originated in the colorectum (*n* = 27), as tumors of unknown origin (*n* = 21), or in the pancreas (*n* = 14) (Table 1). Regarding the site of metastasis, the liver was the predominant organ for both NET G3 (*n* = 13) and NEC (*n* = 26).

Somatostatin receptor type 2 (SSTR2) expression was analyzed in 15 NET G3 and 37 NEC cases and found positive or partially positive in 10/15 (67%) of informative NET G3 and 30/37 (81%) of informative NEC lesions.

When examining the NET G3 group specifically, seven patients stood out with a shorter survival time of less than 10 months. All 7/7 (100%) of these patients presented with extensive liver metastases. In comparison, among those with longer survival, 6/9 (66.7%) also had liver metastases, 2/9 (22.2%) had lymph node metastases, and 1/9 (11.1%) had a small intestinal metastasis. When comparing the Ki-67 labeling index, the short-term survivors showed a median of 28% (range 21–70%), whereas those with longer survival showed a median of 31% (range 22.6–43.7%) (*P* = 0.958).

Regarding growth patterns, all NET G3 tumors displayed an organoid pattern (100%), while a subset also showed a solid growth component (43.8%). Among the short-term survivors, 3/7 (42.9%) displayed a solid pattern, which was comparable to 4/9 (44.4%) among those with longer survival.

With few exceptions, most patients did not receive any treatment before the diagnostic samples were obtained. Among those who received treatment before sampling, the majority experienced disease progression during therapy. Data regarding treatment modalities are summarized in Table 1. Briefly, most NET G3 (*n* = 15, 94%) and NEC (*n* = 48, 71%) patients received adjuvant chemotherapy. Only one NET G3 patient (*n* = 1, 6.25%) received external radiation therapy, compared with 13 (19%) NEC patients. Radionuclide therapy was more frequent among NET G3 cases, both before (*n* = 2, 12.5%) and after (*n* = 4, 25%) sampling, compared with NEC cases (*n* = 3, 4.4% and *n* = 6, 6%, respectively).

Clinical data regarding tumor functionality were not available. However, immunohistochemical analyses were performed on a subset of cases in which the clinical information warranted such evaluation. Staining results for gastrin, insulin, glucagon, somatostatin, pancreatic polypeptide (PP), and serotonin were recorded. Most cases lacked significant immunohistochemical reactivity. In summary, gastrin showed positive staining in approximately 5% of tumor cells in two NEC cases, but in none of the NET G3 cases. Regarding insulin, one NET G3 and one NEC case showed ‘focal’ and ‘minimal’ immunoreactivity, respectively. Glucagon and PP were negative in all cases. Somatostatin was diffusely positive in one PanNET G3 case and showed reactivity in 10–20% of tumor cells in one PanNEC. Serotonin showed diffuse immunoreactivity in one small intestine NET G3, partial reactivity in one GI-NET G3 and one CUP-NET G3, and minimal reactivity in two PanNEC cases.

### Equivocal cases

Three cases exhibited overlapping features of both NET G3 and NEC, making clear classification challenging. These cases are detailed in Table 3. Since they did not fit definitively into either category, these cases were classified as ‘equivocal’ and their features are presented separately. Case #85 was a 72-year-old male patient who presented with a pancreatic primary tumor with metastases to the liver. Biopsy from one of these liver metastases showed a well-differentiated cytomorphology, a Ki-67 labeling index of 45%, wild-type IHC pattern for p53, retained RB1 and DAXX, and loss of ATRX expression, all arguing for a diagnosis of NET G3. On the other hand, the biopsy showed a solid growth pattern and geographical necrosis, arguing in favor of NEC. Case #86 was a 79-year-old male patient who presented with a pancreatic primary tumor from which a biopsy was performed. The biopsy displayed well-differentiated cytomorphology, a Ki-67 labeling index of 50%, and no tumor necrosis, all arguing in favor of NET G3. On the other hand, the tumor displayed a solid pattern of growth with aberrant p53 staining, arguing for NEC. Case #87 was a 73-year-old female patient who presented with a cancer of unknown primary (CUP) with liver metastases. Biopsy from a liver metastasis showed large pleomorphic cells in a solid growth pattern with focal necrosis, all arguing for NEC. However, the Ki-67 labeling index was 28%, with p53 and RB1 showing wild-type and retained staining patterns, respectively, all arguing in favor of NET G3. This approach reflects ongoing challenges in the scientific field, where certain lesions may exhibit overlapping characteristics, recently referred to as ‘NEC-like features’ in well-differentiated NETs (16, 17, 18, 20).

**Table 3:** Equivocal cases.

Parameter	Case #85	Case #86	Case #87
Sex	Male	Male	Female
Age	72	79	73
Primary	Pancreas	Pancreas	CUP
Metastatic site	Liver	Not specified	Liver
Type of specimen	Biopsy	Biopsy	Biopsy
Specimen characteristic	Metastasis	Primary	Metastasis
Morphology/growth pattern	PD/solid, only	PD/solid, only	PD/solid, only
Ki-67 index (%)	45	50	28
Necrosis	Present	Absent	Present
p53	Wild-type	Aberrant/mutated	Wild-type
RB1	Retained	Retained	Retained
ATRX	Lost/mutated	Retained	Not specified
DAXX	Retained	Retained	Not specified

CUP, cancer of unknown primary; PD, poorly differentiated; RB1, retinoblastoma protein; ATRX, alpha-thalassemia/mental retardation syndrome X-linked; DAXX, death domain-associated protein.

### Association to clinical outcomes

**Figure 2:**
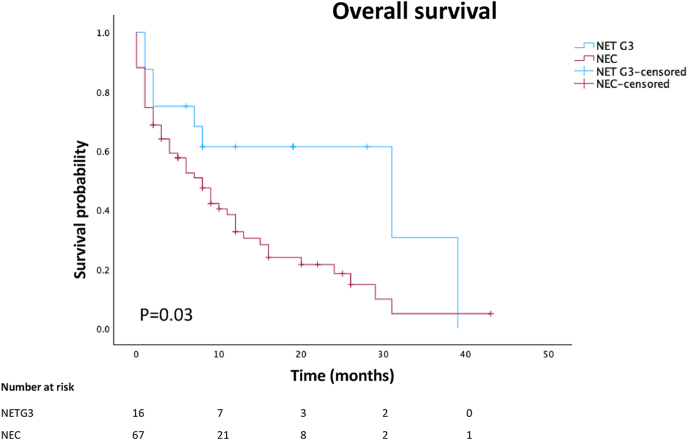
Kaplan–Meier survival analysis comparing neuroendocrine tumor grade 3 (NET G3) and neuroendocrine carcinoma (NEC) (log-rank test, *P* = 0.03).

Kaplan–Meier survival analysis revealed a significant difference in overall survival between NET G3 and NEC patients. Patients with NET G3 exhibited longer survival compared with those with NEC (log-rank test, *P* = 0.03), as demonstrated in Fig. 2. NEC patients had a markedly shorter median survival of 6 months (95% CI: 0–43).

Given the larger patient cohort presented within the NEC group, additional analyses were performed on this tumor group. Using univariate analysis for clinical and histological parameters potentially associated with poorer survival, significant correlations were noted for necrosis (hazard ratio; HR 0.506, 95% CI: 0.265–0.968, *P* = 0.040) and either colorectal primary (HR 0.494, 95% CI: 0.264–0.925, *P* = 0.027) or CUP designation (i.e., unknown primary tumor location) (HR 2.033, 95% CI: 1.109–3.727, *P* = 0.022). However, in multivariate analyses, no parameters remained significant, arguing for confounding factors.

Upon review, 60 of 67 informative NEC cases (89.6%) presented with metastatic disease at diagnosis. Among patients who underwent primary tumor resection (excluding CUP cases), 14 of 18 (77.8%) presented with metastatic disease. All NEC patients who were metastasis-free at diagnosis were alive at last follow-up (100%), whereas 7 of 14 (50%) patients with metastatic disease at the time of primary tumor resection had died by the time of last follow-up. Statistically, metastasis at diagnosis was not significantly associated with mortality among patients who underwent primary surgery (data not shown). There was also no significant association between the presence of necrosis and metastatic status at diagnosis (Fisher’s exact test, *P* = 0.235), indicating that necrosis was not related to disease stage at presentation.

To determine whether the availability of larger tissue samples in resection specimens (as compared with biopsies) might have influenced the detection of necrosis, we compared its frequency between these groups. Necrosis was identified in 13 of 13 (100%) resected cases, a higher proportion than in 22 of 42 (52.4%) biopsy cases (Fisher’s exact test, *P* = 0.002). Patients who underwent surgical resection showed a trend toward improved survival compared with those diagnosed by biopsy alone (Cox regression HR 0.498, 95% CI: 0.233–1.065, *P* = 0.072; log-rank test, *P* = 0.058). This observation suggests that the higher frequency of necrosis in surgical specimens may partly reflect sampling differences rather than underlying biological behavior. When comparing patients who had received neoadjuvant treatment before biopsy or surgery with those who had not, necrosis was observed in 9 of 13 (69.2%) and 26 of 42 (61.9%) cases, respectively (Fisher’s exact test, *P* = 0.749), indicating that neoadjuvant treatment did not significantly affect the presence of necrosis.

## Discussion

This study highlights the histopathological differences between NET G3 and NEC lesions, emphasizing the importance of subclassification of tumors with a Ki-67 index >20% into these distinct entities. The significant disparity in overall survival between NET G3 and NEC patients supports this distinction, with NET G3 lesions displaying a markedly better prognosis. These findings validate the emerging concept of NET G3 as a unique tumor entity and underscore the necessity of its recognition in routine diagnostics.

The value of immunohistochemical markers and histological growth patterns in differentiating NET G3 from NEC was evident in this study. Aberrant p53 and RB1 staining patterns were significantly more common in NECs than NET G3 lesions, corroborating their utility as diagnostic tools. Notably, in this study, immunohistochemical loss of RB1 appears to support the diagnosis of NEC rather than NET G3 to a greater extent than either SSTR2 or p53 alterations. While 1 of 11 NET G3 cases showed aberrant p53 immunostaining, none demonstrated RB1 loss, consistent with previous findings (21). In addition, the observation that NET G3 tumors frequently displayed an organoid growth pattern, while NECs were more likely to exhibit a solid growth pattern and necrosis, reinforces the diagnostic relevance of histological evaluation.

In our cohort, the univariate association between tumor necrosis and improved survival among NEC patients likely reflects a sampling bias rather than a true biological effect. Necrosis was detected more frequently in surgically resected specimens than in biopsies, a finding that can be attributed to the larger volume of tissue available for histologic evaluation in resection specimens. Since patients eligible for primary resection generally represent a more favorable clinical subset – often with lower metastatic burden and better overall condition – the apparent survival advantage associated with necrosis is likely confounded by this selection.

The analyses of the NEC cohort identified tumor origin as a significant factor associated with variations in patient survival. Specifically, patients with confirmed colorectal primaries demonstrated improved outcomes, with a protective effect observed in univariate analysis. In contrast, patients with CUP exhibited significantly poorer survival. These findings suggest that tumor origin plays a critical role in the prognosis of NEC patients. The better outcomes for colorectal NECs may reflect distinct biological behavior or different treatment responses, whereas the poor prognosis for CUP likely underscores the challenges associated with managing tumors of unknown origin, including delayed or suboptimal treatment (22, 23, 24, 25). Alternatively, the frequently poor differentiation seen in CUP cases may itself be a risk factor for poor prognosis.

A notable finding in our study was the unexpectedly high rate of SSTR2 expression in NEC lesions, with 81% displaying positive or partially positive staining. While SSTR2 positivity is typically associated with well-differentiated NETs, its prevalence in NECs raises intriguing possibilities (26, 27, 28, 29, 30). This observation suggests that a significant proportion of NECs may be visualized using somatostatin receptor-based imaging and could thus potentially benefit from DOTATOC-based somatostatin analog therapies. Future studies are needed to explore the therapeutic implications of this finding and to assess the clinical efficacy of these approaches in NEC patients. A limitation of our study is that only a small subset of cases was stained for SSTR2, primarily due to the limited availability of tissue material. In routine diagnostics, the responsible pathologist often needs to prioritize immunohistochemical stains essential for determining neuroendocrine differentiation, tumor grade, and primary site of origin before SSTR2 staining can be considered (31).

Three cases in our cohort exhibited overlapping features of NET G3 and NEC, making definitive classification challenging. These equivocal cases highlight the potential for a spectrum between these entities, as previously suggested (20). Such overlap may reflect shared oncogenic pathways or transitional states between well-differentiated and poorly differentiated neoplasms. Recognizing and characterizing these ambiguous cases is crucial for advancing the understanding of neuroendocrine neoplasms and refining diagnostic criteria.

Our study has several limitations that should be acknowledged. While some novel observations were identified, the results largely align with existing knowledge. Moreover, the absence of molecular profiling limits the depth of biological interpretation. This highlights a practical challenge in the clinical management of these tumors, where only limited biopsy material is often available. Furthermore, although the cases were consecutively collected, the study was not a prospective interventional trial with predefined treatment algorithms. Treatment allocation followed routine clinical practice and was not standardized, which may have introduced variability between NET G3 and NEC patients. Finally, the small sample size – particularly for NET G3 – and relatively short follow-up period restrict the strength of survival-related conclusions.

Despite these limitations, our findings underscore the critical need for precise subclassification of high-grade neuroendocrine neoplasms. The difference in survival between NET G3 and NEC patients reinforces the clinical utility of distinguishing these entities, with established implications for prognosis and treatment strategies. The diagnostic role of p53 and RB1 immunohistochemistry, and the histological growth pattern assessment, reinforces the pathological toolkit for accurate classification. This study provides evidence supporting the clinical routine subdivision of high-grade neuroendocrine neoplasms into NET G3 and NEC, offering insights into their distinct histopathological profiles.

## Declaration of interest

The authors declare that there is no conflict of interest that could be perceived as prejudicing the impartiality of the work reported.

## Funding

This study was financially supported by the Swedish Cancer Society (23 0616 SCIA).

## Author contribution statement

CCJ conceived and designed the study, developed the main conceptual framework, and provided financial support. ZA conducted the histopathological and immunohistochemical evaluations, drafted the manuscript, and created the figures, with input from CCJ and LSH. AS and JZ were responsible for clinical follow-up of the patient cohorts. Statistical analyses were performed by ZA and LSH. All authors contributed to and approved the final version of the manuscript.
